# Biomarkers of Cardiac Injury, Renal Injury, and Inflammation Are Strong Mediators of Sex-Associated Death in COVID-19

**DOI:** 10.3389/fcvm.2022.809997

**Published:** 2022-04-25

**Authors:** Heidi S. Lumish, Eunyoung Kim, Caitlin Selvaggi, Tingyi Cao, Aakriti Gupta, Andrea S. Foulkes, Muredach P. Reilly

**Affiliations:** ^1^Division of Cardiology, Columbia University, New York, NY, United States; ^2^Biostatistics Center, Massachusetts General Hospital, Boston, MA, United States; ^3^Department of Medicine, Harvard Medical School, Boston, MA, United States; ^4^Department of Biostatistics, Harvard T. H. Chan School of Public Health, Boston, MA, United States; ^5^Division of Interventional Cardiology, Cedars-Sinai Medical Center, Los Angeles, CA, United States; ^6^Irving Institute for Clinical and Translational Research, Columbia University, New York, NY, United States

**Keywords:** biomarkers, myocardial injury, SARS-CoV-2, sex differences, inflammation

## Abstract

**Background:**

Studies examining outcomes among individuals with COronaVIrus Disease 2019 (COVID-19) have consistently demonstrated that men have worse outcomes than women, with a higher incidence of myocardial injury, respiratory failure, and death. However, mechanisms of higher morbidity and mortality among men remain poorly understood. We aimed to identify mediators of the relationship between sex and COVID-19-associated mortality.

**Methods:**

Patients hospitalized at two quaternary care facilities, New York Presbyterian Hospital (CUIMC/NYPH) and Massachusetts General Hospital (MGH), for SARS-CoV-2 infection between February and May 2020 were included. Five independent biomarkers were identified as mediators of sex effects, including high-sensitivity cardiac troponin T (hs-cTNT), high sensitivity C-reactive protein (hs-CRP), ferritin, D-dimer, and creatinine.

**Results:**

In the CUIMC/NYPH cohort (*n* = 2,626, 43% female), male sex was associated with significantly greater mortality (26 vs. 21%, *p* = 0.0146) and higher peak hs-cTNT, hs-CRP, ferritin, D-dimer, and creatinine (*p* < 0.001). The effect of male sex on the primary outcome of death was partially mediated by peak values of all five biomarkers, suggesting that each pathophysiological pathway may contribute to increased risk of death in men. Hs-cTnT, creatinine, and hs-CRP were the strongest mediators. Findings were highly consistent in the MGH cohort with the exception of D-dimer.

**Conclusions:**

This study suggests that the effect of sex on COVID-19 outcomes is mediated by cardiac and kidney injury, as well as underlying differences in inflammation and iron metabolism. Exploration of these specific pathways may facilitate sex-directed diagnostic and therapeutic strategies for patients with COVID-19 and provides a framework for the study of sex differences in other complex diseases.

## Introduction

Across numerous studies of COronaVIrus Disease 2019 (COVID-19), men have had consistently worse rates of severe outcomes than women, with higher rates of cardiac injury, respiratory failure, shock, intensive care unit (ICU) admission, and death (1–3). This sex-related difference in outcomes has been confirmed in cohorts from China (4), Italy (5), and the United States (2, 6). There are multiple mechanisms hypothesized to contribute to this sex difference in COVID-19 outcomes – for example, sex-related factors that affect disease susceptibility, including differences in smoking and drinking habits, rates of handwashing, and social obligations (7). Men are also known to have higher rates of baseline co-morbidities, including hypertension and cardiovascular disease (8), and they may be more susceptible to the effects of age and co-morbidities than women (9). However, prior studies have demonstrated that the sex-related difference in outcomes is not entirely accounted for by a difference in baseline co-morbidities (10). Hormonal and genetic factors are also thought to play a role in disease pathogenesis. Sex hormones have been shown to modify inflammatory pathways and affect the regulation of angiotensin converting enzyme 2 (ACE2), which mediates Severe Acute Respiratory Syndrome Coronavirus-2 (SARS-CoV-2) entry into cells (11–13). Genes conferring immunity are located on the X chromosome, some of which escape X-inactivation leading to a dose-related difference in the gene effect between men and women (11, 12). There are likely other important pathophysiological determinants of sex differences that are incompletely understood.

Downstream of social, clinical, hormonal and genetic processes, there are multiple biological pathways driving COVID-19 outcomes that may be mechanistically important and therapeutically tractable in the described sex differences. Several studies have demonstrated a sex difference in the relationship between various biomarkers and COVID-19 outcomes. Candidate biomarkers are implicated in immune response, inflammatory pathways, and coagulation pathways, as well as end organ dysfunction (9, 11, 14, 15). Specifically, men have been shown to have higher incidence of myocardial injury, as measured by troponin elevation (3). However, to date there has been no systematic examination of biomarkers of these pathophysiological processes and their relative effects as mediators of the sex difference in COVID-19 outcomes. Mediation analysis, our novel focus in this paper, aims to inform whether a biomarker (of a pathologic process) is in the causal pathway between an exposure (e.g., sex) and outcome (e.g., death) whereas the assessment of modification, a focus of prior studies (15–17), examines whether the exposure interacts with a biomarker in its association with the outcome.

In this study, we investigated potential pathophysiological biomarker mediators of the effect of sex on COVID-19 outcomes, to better understand the potential mechanisms and therapeutic implications for increased risk of poor outcomes in men. We evaluated 15 candidate blood biomarkers of biological pathways perturbed in COVID-19 as potential mediators of sex differences in COVID-19 outcomes, including markers of cardiac injury, inflammation, iron metabolism and coagulation, as well as renal and liver injury.

## Methods

### Study Populations

The study population included a total of 4,017 patients hospitalized for SARS-CoV-2 infection between February and May 2020 at two independent quaternary care facilities, Columbia University Irving Medical Center/New York Presbyterian Hospital (CUIMC/NYPH) and Massachusetts General Hospital (MGH).

#### CUIMC/NYPH COVID-19 Cohort

The CUIMC/NYPH COVID-19 cohort includes 2,626 adult patients (≥18 years of age) who were hospitalized at CUIMC and the Allen Hospital sites of NYPH between February 1 and May 12, 2020, with positive SARS-CoV-2 reverse transcriptase-polymerase chain reaction testing of nasopharyngeal or oropharyngeal specimens (18). Patients who were admitted for <24 h were excluded from the analysis. Patients were followed until discharge, death, or the end of study follow-up on June 11, 2020. Patient data were identified in the electronic health record (EHR) by using the institution's clinical data warehouse, which included information on individuals who receive care at CUIMC/NYPH. Analysis was based on index hospitalization. Clinical comorbidities including hypertension, diabetes, coronary artery disease (CAD), heart failure, stroke or transient ischemic attack, atrial arrhythmias (atrial fibrillation, atrial flutter, and supraventricular tachycardia), chronic lung disease, chronic kidney disease, and chronic liver disease, were identified using ICD-10 medical billing codes (**Supplementary Table S1**). Cancer was defined by an automated search of the EHR for the terms “cancer,” “carcinoma,” “malignancy,” “malignant,” “neoplasm,” “-noma,” or “blastoma,” excluding those with the terms “screen” or “hypertension.” Obesity was defined as body mass index (BMI) ≥30 at the time of index hospitalization. The primary outcome was in-hospital mortality within 30 days of admission. Peak biomarker values were defined over the duration of hospitalization.

#### MGH COVID-19 Patient Registry

A replication analysis was performed using retrospective data on 1,391 individuals from the MGH COVID-19 Patient Registry (19, 20). All patients were hospitalized between March 11, 2020 and May 31, 2020 and tested positive for SARS-CoV2. Demographic information, comorbid conditions, medications, laboratory tests, and clinical outcomes at index hospitalization were manually extracted from electronic health records. The primary outcome was death within 28 days of presentation to care, defined as first contact with a health care provider due to COVID-19-related symptoms. Peak biomarker values were defined over the duration of hospitalization within 28 days of index date.

### Statistical Methods

Baseline characteristics were summarized for the overall cohort and stratified by sex for both the CUIMC/NYP and MGH registries. Unadjusted two-sided tests of proportions (or means) were used to compare baseline characteristics for male and female patients.

In the CUIMC/NYPH data, we evaluated 15 biomarkers of pathways perturbed in COVID-19 as potential mediators of sex differences in COVID-19 outcomes. Of these, two (IL-6, lactate) were excluded because of >50% missing data, and four (albumin, ALC, ESR, platelets) were excluded because their peak values were not associated with sex. Although associated with sex, WBC and automated lymphocytes were not reported as main findings because they were correlated and redundant with hs-CRP, which had stronger mediation effects (**Supplementary Table S2**). Hepatic injury markers (AST and ALT) were excluded because of race/ethnicity interactions in their association with death, limiting statistical power for race/ethnicity-specific mediation effects (**Supplementary Table S2**). Details on all additional biomarkers that were screened but not presented as primary findings are given in **Supplementary Table S2**. Thus, five biomarkers representing distinct biological pathways – hs-cTNT, hs-CRP, ferritin, creatinine, and D-dimer – were analyzed and presented here for their mediation of sex effects on death during index hospitalization. Each of these had peak values that were significantly associated with sex, had observed data in >50% of individuals, and represented distinct pathophysiological processes. Peak biomarker values were natural log transformed and standardized prior to inclusion in models.

We applied the causal mediation analysis approach described by Imai et al. (21) and as we applied in Foulkes et al. (22) which uses the results of three models to determine the proportion of the association between sex and severe outcomes that is mediated by the biomarker: (1) A Total Effect Model using a logit link with death (Y) as the outcome and sex (T) as a predictor variable, where the biomarker (M) is not included in the model; (2) A Mediator Model using an identify link with peak biomarker as the outcome and sex as a predictor; and (3) An Outcome Model using a logit link with death as the outcome and both sex and peak biomarker as predictor variables. All models were initially conditioned on age, sex, obesity, race/ethnicity and number of biomarker measurements. The reported odds ratios (ORs) are computed in the same way as using standard statistical model fitting procedures.

The average proportion mediated and corresponding *p*-value were reported for each biomarker. Models were fitted overall based on data from adult patients (≥18 years of age) using the peak biomarker value for each patient. Analysis used a complete case analysis for each biomarker separately assuming data were missing completely at random. Summary level data on characteristics of patients with and without missing data are provided (**Supplementary Table S3**). Primary analysis was based on data derived from the CUIMC/NYP cohort. Replication analysis was based on the MGH cohort. To explore mediation effects that might differ by menopausal status in women, we performed secondary analyses of age strata (≥ 50 vs. <50 years of age) designed as a surrogate for pre- and post-menopausal status in women. Additional sensitivity analyses in the CUIMC/NYP cohort were performed to check the consistency of our conclusions when (1) adjusting for additional potential confounders (coronary artery disease, chronic kidney disease, lung disease, hypertension, type 2 diabetes mellitus, cancer, heart failure, and stroke) in the multivariable models, (2) restricting to individuals with complete data for all variables (*N* = 1,688). Analyses were completed using R version 3.5.0. Mediation analysis was performed using the R package “mediation.”

### Human Subjects and IRB Approvals

The CUIMC IRB approved this study (#AAAS9835) and waived the requirement for obtaining informed consent. The Partners HealthCare Institutional Review Board (IRB) (#2020P000829) approved collection of curated data based on comprehensive manual chart reviews and data extractions from EHRs on patients who receive care through the Mass General Brigham (MGB, formerly Partners) system.

## Results

### Baseline Characteristics of the CUIMC/NYP COVID-19 Cohort

Baseline clinical, demographic, and laboratory findings, overall and stratified by sex, are shown in Table 1. Median age was 66 (IQR 54, 77) years. Women accounted for 43% of the study cohort, and women were older (69 [57, 80] vs. 64 [53, 75], *p* < 0.001) and more obese (BMI 29.0 [24.9, 34.1] vs. 27.5 [24.4, 31.5], *p* < 0.001) than men. There were no significant differences in baseline statin and angiotensin-converting enzyme inhibitors (ACEi) or angiotensin receptor blocker (ARB) use between men and women. Men had a trend toward higher rates of baseline CAD, though this was not statistically significant, and women had significantly higher rates of hypertension (58 vs. 52%, *p* = 0.0047) and lung disease (23 vs. 14%, *p* < 0.001). There were no sex differences in the baseline proportions of patients with diabetes mellitus, chronic kidney disease (CKD), cancer, heart failure or stroke (Table 1). On admission, compared to women, men had significantly higher hs-CRP, creatinine, hs-cTnT levels and especially ferritin. There was no sex difference in D-dimer levels at the time of admission (Table 1).

**Table 1:** Clinical characteristics and admission labs overall and by sex in the CUIMC/NYP COVID-19 cohort.

	**Overall (*N* = 2,626)**	**Male (*N* = 1,497)**	**Female (*N* = 1,129)**	*P*-value^*^
**Presentation to care**				
Age in years (median [IQR])	66 (54, 77)	64 (53, 75)	69 (57, 80)	<0.001
Age ≥65 years	1,420/2,626 (0.54)	748/1,497 (0.50)	672/1,129 (0.60)	<0.001
White/non-Hispanic	237/2,626 (0.09)	139/1,497 (0.09)	98/1,129 (0.09)	0.6406
Black/non-Hispanic	320/2,626 (0.12)	180/1,497 (0.12)	140/1,129 (0.12)	0.8169
Hispanic	1,314/2,626 (0.50)	747/1,497 (0.50)	567/1,129 (0.50)	0.9015
Other	755/2,626 (0.29)	431/1,497 (0.29)	324/1,129 (0.29)	0.9932
Fever	604/2,624 (0.23)	371/1,497 (0.25)	233/1,127 (0.21)	0.0152
Body mass index (Median [IQR])	28.02 (24.60, 32.66)	27.46 (24.36, 31.46)	28.96 (24.89, 34.13)	<0.001
On statins	951/2,626 (0.36)	519/1,497 (0.35)	432/1,129 (0.38)	0.0634
On ACEi or ARBs^†^	442/2,626 (0.17)	238/1,497 (0.16)	204/1,129 (0.18)	0.1559
**Co-morbidities**				
Obesity^‡^	794/2,113 (0.38)	393/1,219 (0.32)	401/894 (0.45)	<0.001
Coronary artery disease	329/2,626 (0.13)	204/1,497 (0.14)	125/1,129 (0.11)	0.0576
Hypertension	1,430/2,626 (0.54)	779/1,497 (0.52)	651/1,129 (0.58)	0.0047
Diabetes mellitus type 2	968/2,626 (0.37)	553/1,497 (0.37)	415/1,129 (0.37)	0.9561
Chronic kidney disease	370/2,626 (0.14)	219/1,497 (0.15)	151/1,129 (0.13)	0.3908
Lung disease	463/2,626 (0.18)	207/1,497 (0.14)	256/1,129 (0.23)	<0.001
Cancer	261/2,626 (0.10)	155/1,497 (0.10)	106/1,129 (0.09)	0.4517
Heart failure	275/2,626 (0.10)	149/1,497 (0.10)	126/1,129 (0.11)	0.3494
Stroke	225/2,626 (0.09)	130/1,497 (0.09)	95/1,129 (0.08)	0.8620
**Admission labs (median [IQR])** ^§^				
hs-CRP (mg/L; *n* = 2,414)	118.46 (56.79, 205.18)	130.48 (63.66, 215.36)	105.79 (45.40, 184.04)	<0.001
D-Dimer (ng/mL; *n* = 2,179)	1,510 (830, 3,290)	1,490 (800, 3,440)	1,520 (873, 3,170)	0.6767
Ferritin (ng/mL; *n* = 2,391)	702.6 (345.40, 1,293)	870.4 (457.80, 1584.50)	479.4 (238.80, 929.60)	<0.001
Creatinine (mg/dL; *n* = 2,609)	1.07 (0.81, 1.64)	1.17 (0.91, 1.75)	0.92 (0.70, 1.48)	<0.001
hs-cTnT (ng/L; *n* = 2,402)	17 (8, 42)	19 (9, 43)	16 (8, 39)	0.0028

* *P-values correspond to a two-sample test of proportions (for categorical variables) or Wilcoxon rank sum tests (for numeric variables) comparing corresponding characteristics of male vs. female patients*;

† *ACEi, angiotensin-converting enzyme inhibitors; ARB, angiotensin receptor blockers*;

‡ *Obesity is defined as BMI ≥30 and is missing for 513 patients*;

§ *Admission labs - recorded within +/-3 days of hospital admission*.

*IQR, interquartile range; hs-CRP, high sensitivity C-reactive protein; hs-cTNT, high sensitivity cardiac Troponin T*.

### Unadjusted Analyses of Biomarkers and Outcome by Sex in the CUIMC/NYP COVID-19 Cohort

Peak values for all five biomarkers were significantly higher in men than in women, as shown in Table 2. The most notable sex difference was in median peak ferritin level, which was 1184.5 (IQR 621, 2,269) in men as compared to 615.5 (IQR 295, 1,307) in women. Mortality at 30 days was significantly higher in men than in women (26 vs. 21%, *p* = 0.0146, Table 2).

**Table 2:** Peak laboratory values and outcomes overall and by sex in the CUIMC/NYP COVID-19 cohort.

	**Overall (*N* = 2,626)**	**Male (*N* = 1,497)**	**Female (*N* = 1,129)**	*P*-value^*^
**Peak labs (median [IQR])** ^†^				
hs-CRP (mg/L; *n* = 2,416)	167.12 (83.80, 281.45)	185.11 (101.11, 293.01)	143.99 (65.80, 261.38)	<0.001
D-Dimer (ng/ml; *n* = 2,180)	2,565 (1060, 9805)	2,790 (1060, 12260)	2,230 (1050, 7463)	0.0016
Ferritin (ng/ml; *n* = 2,393)	931.40 (437.90, 1,934)	1,184.5 (620.70, 2,269)	615.5 (295.40, 1,307)	<0.001
Creatinine (mg/dL; *n* = 2,609)	1.34 (0.92, 2.71)	1.49 (1.05, 3.07)	1.11 (0.80, 2.28)	<0.001
hs-cTnT (ng/L; *n* = 2,402)	26 (10, 79)	29 (11, 84)	24 (9, 67)	<0.001
**Follow-up (30 day) outcomes**				
Ventilator or death	908/2,626 (0.35)	555/1,497 (0.37)	353/1,129 (0.31)	0.0022
Ventilator^‡^	559/2,626 (0.21)	365/1,497 (0.24)	194/1,129 (0.17)	<0.001
Death	623/2,626 (0.24)	382/1,497 (0.26)	241/1,129 (0.21)	0.0146

* *P-values correspond to a two-sample test of proportions (for categorical variables) or Wilcoxon rank sum tests (for numeric variables) comparing corresponding characteristics of male vs. female patients*;

† *Peak labs – high sensitivity C-reactive protein (hs-CRP), high sensitivity cardiac Troponin T (hs-cTnT)*

‡ *349 patients died without being on ventilator*.

*IQR, interquartile range; hs-CRP, high sensitivity C-reactive protein; hs-cTNT, high sensitivity cardiac Troponin T*.

### Mediation Effects in the CUIMC/NYP COVID-19 Cohort

**Figure 1:**
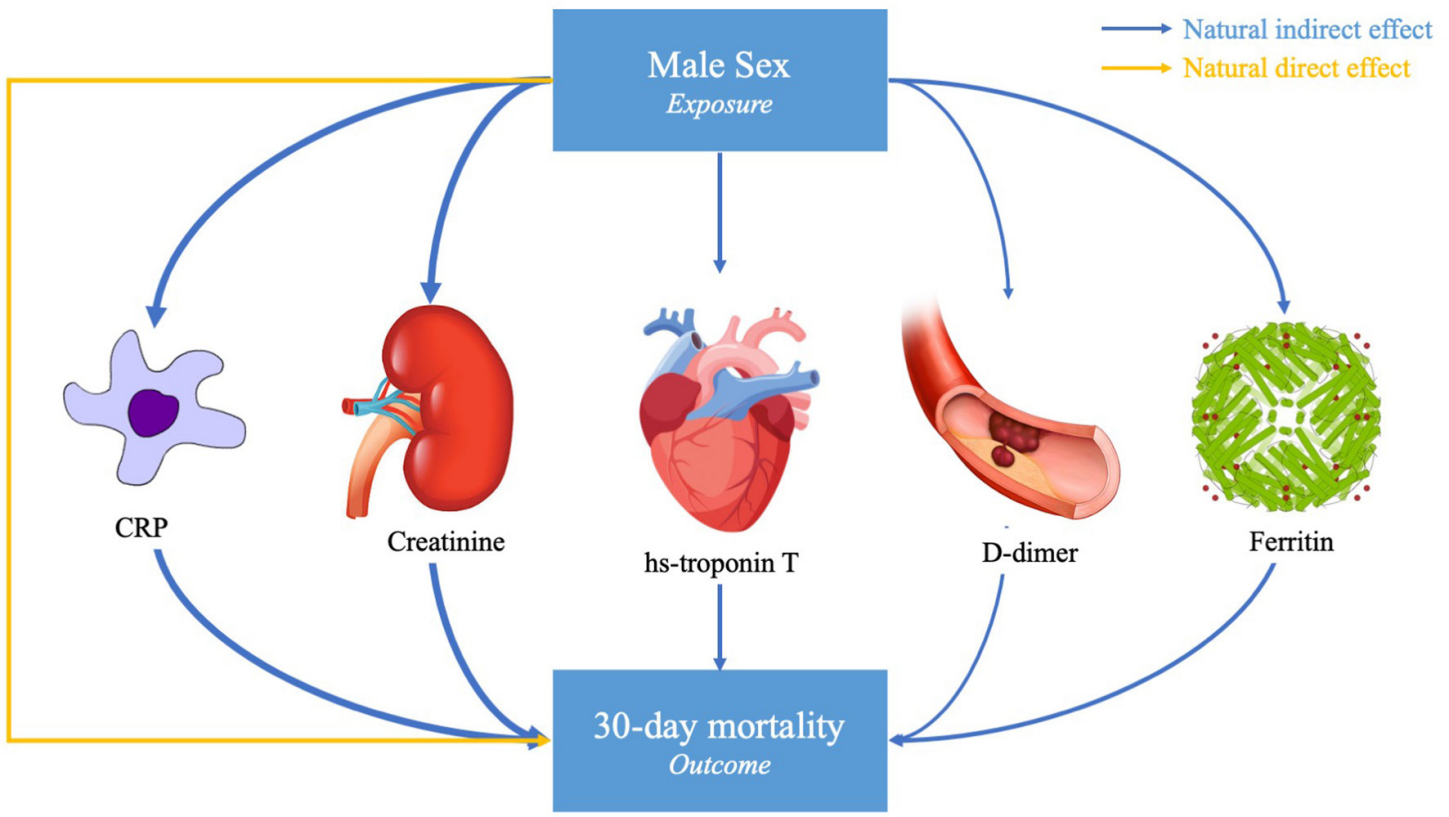
Causal mediation model. This study assessed the degree to which peak serum biomarkers mediated the association between male sex and death due to SARS-CoV-2 infection in hospitalized patients. Of the fifteen biomarkers tested, five were significant mediators of the association between male sex and 30-day mortality, with varying proportion mediated as represented by the arrow thickness. Potential confounders accounted for in the primary analysis included age, sex, race/ethnicity, and number of biomarker measurements. The proportion mediated is given by the Natural Indirect Effect (NIE) divided by the Total Effect [NIE + Natural Direct Effect (NDE)].

In adjusted models, male sex was a significant predictor of death at 30 days (OR ~2.0 depending on the specific sample of patients with available biomarker data, *p* < 0.001, Table 3). Male sex was also a significant predictor of the peak hs-CRP, ferritin, D-dimer, hs-cTnT, and creatinine (*p* < 0.001, Table 3). The effect of sex on the primary outcome of death was partially attenuated after adjustment for each of the five peak biomarker values, suggesting a potential mediation effect for each. The proportion mediated was significantly different than 0 for all of the biomarkers. The estimated proportion mediated was greatest for hs-cTnT (0.45, *p* < 0.001), hs-CRP (0.42, *p* < 0.001), and creatinine (0.35, *p* < 0.001) and lowest for D-dimer (0.22, *p* < 0.001, Table 3, Figure 1).

**Table 3:** Primary mediation analyses of peak values^*^ of biomarkers in CUIMC/NYP COVID-19 cohort.

	**Total effect model^†^ outcome: death**	**Mediator model^†^ outcome: biomarker**	**Outcome model**^†^ **outcome: death**		**Proportion mediated**
	**OR (sex)**	**Estimate (sex)**	**OR (sex)**	**OR (biomarker)**	
**hs-CRP**					
All (*n* = 1,978)	2.00 (*p* <0.001)	0.285 (*p* <0.001)	1.71 (*p* <0.001)	-^‡^	0.42 (*p* <0.001)
Obese (*n* = 748)	2.00 (*p* = 0.001)	0.204 (*p* = 0.002)	1.87 (*p* = 0.005)	8.24 (*p* <0.001)	0.42 (*p* <0.001)
Non-obese (*n* = 1,230)	2.00 (*p* <0.001)	0.329 (*p* <0.001)	1.62 (*p* = 0.003)	2.80 (*p* <0.001)	0.42 (*p* <0.001)
**Ferritin**					
All (*n* = 1,070)	2.33 (*p* <0.001)	0.474 (*p* <0.001)	2.04 ( *p* = 0.002)	-^‡^	0.33 (*p* <0.001)
Obese (*n* = 483)	1.96 (*p* = 0.046)	0.510 (*p* <0.001)	1.57 ( *p* = 0.204)	2.62 (*p* <0.001)	0.51 ( *p* = 0.010)
Non-obese (*n* = 587)	2.64 (*p* = 0.001)	0.456 (*p* <0.001)	2.47 ( *p* = 0.003)	1.92 (*p* <0.001)	0.24 (*p* <0.001)
**D-dimer**					
All (*n* = 1,814)	2.03 (*p* <0.001)	0.207 (*p* <0.001)	1.84 ( *p* <0.001)	-^‡^	0.22 (*p* <0.001)
*≥ 65 yrs* (*n* = 945)	1.88 (*p* <0.001)	0.165 (*p* = 0.010)	1.76 (*p* <0.001)	2.13 (*p* <0.001)	0.18 (*p* = 0.008)
* <65 yrs* (*n* = 869)	2.44 (*p* = 0.001)	0.205 (*p* = 0.005)	2.17 (*p* = 0.007)	3.35 (*p* <0.001)	0.25 (*p* = 0.006)
**Creatinine**					
All (*n* = 2,106)	1.98 (*p* <0.001)	0.430 (*p* <0.001)	1.52 (p=0.001)	2.24 (*p* <0.001)	0.45 (*p* <0.001)
**hs-Troponin T**					
All (*n* = 1,954)	2.05 (*p* <0.001)	0.301 (*p* <0.001)	1.70 ( *p* <0.001)	2.55 (*p* <0.001)	0.35 (*p* <0.001)

* *Peak biomarker level was determined based on all measurements*.

*All values were natural log transformed and standardized for analysis*;

† *All models included terms for sex and were adjusted for age, obesity, race/ethnicity, and the number of biomarker measurements*.

*The outcome model included both sex and the biomarker as predictor variables*;

‡ *The outcome model included a biomarker by obesity/age interaction and therefore the main effect of the biomarker was not reported here*.

*OR, odds ratio; hs-CRP, high sensitivity C-reactive protein; hs-Troponin T, high sensitivity cardiac Troponin T*.

There were interaction effects with biomarkers in the outcome model for hs-CRP and ferritin with obesity and for D-dimer with age, and therefore stratified analyses are included in these cases. In the obesity (BMI >30) stratified analyses for hs-CRP, the estimated proportion mediated remained the same for obese and non-obese patients (0.42, *p* < 0.001 for each). While ferritin remained a significant mediator of the effect of sex on COVID-19 outcomes after stratifying by obesity, the estimated proportion mediated was larger among obese patients (0.51, *p* = 0.010) as compared to non-obese patients (0.24, *p* < 0.001). For D-dimer, the estimated proportion mediated was slightly lower in the older strata (0.18 for >65 years vs. 0.25 for age <65 years, Table 3). In secondary age-stratified analyses (≥ or < age 50), a surrogate for menopausal status in women, the mediation effects of each biomarker were largely consistent across younger vs. older age categories. However, hs-CRP had a greater proportion mediated for age <50 years (0.59) as compared to those with age ≥ 50 years (0.35), and ferritin had a trend toward a greater proportion mediated in those aged <50 (0.57 vs. 0.42, **Supplementary Table S4**).

### Replication Analyses in the MGH COVID-19 Patient Registry

Replication analysis was performed in the MGH registry. The clinical characteristics, laboratory values, and outcomes overall and by sex in the MGH cohort are shown in Supplementary Tables S5, S6. Similar to the CUIMC/NYP cohort, women were more obese than men (BMI 29.8 [25.6, 35.0] vs. 28.6 [25.2, 32.9], *p* = 0.016) and had significantly more lung disease (33 vs. 27%, *p* = 0.030). While in the CUIMC/NYP cohort men had a trend toward higher rates of CAD, in the MGH cohort men had significantly more CAD and were also more likely to be smokers. In unadjusted analysis, peak values for all five biomarkers were significantly higher in men than in women, consistent with the CUIMC/NYP cohort. Also in unadjusted analysis and similar to the CUIMC/NYP cohort, men in the MGH cohort had significantly higher rates of the primary endpoint of death.

The results of the mediation analysis of the five candidate biomarkers in the MGH cohort are shown in Table 4. CRP, ferritin, creatinine, and hs-cTnT were all significant mediators of the effect of sex on COVID-19-related mortality. Stratified analyses by obesity for CRP and ferritin had similar patterns to the CUIMC/NYP cohort with no difference in proportion mediated between obese and non-obese for CRP but a greater proportion mediated for obese with ferritin (0.51 vs. 0.24). While D-dimer was a significant mediator in the CUIMC/NYP cohort, it was not a significant mediator in the MGH registry data.

**Table 4:** Replication mediation analyses of peak values^*^ of biomarkers in MGH cohort.

	**Total effect model^†^ outcome: death**	**Mediator model^†^ outcome: biomarker**	**Outcome model**^†^ **outcome: death**		**Proportion mediated**
	**OR (sex)**	**Estimate (sex)**	**OR (sex)**	**OR (biomarker)**	
**hs-CRP**					
All (*n* = 1,088)	2.24 (*p* <0.001)	0.102 (*p* = 0.058)	2.09 (*p* = 0.002)	-^‡^	0.20 (*p* = 0.048)
Obese (*n* = 491)	1.92 (*p* = 0.051)	0.080 (*p* = 0.277)	1.78 (*p* = 0.123)	9.07 (p <0.001)	0.22 (*p* = 0.284)
Non-obese (*n* = 597)	2.44 (*p* = 0.002)	0.144 (*p* = 0.065)	2.18 (*p* = 0.012)	4.64 (*p* <0.001)	0.22 (*p* = 0.062)
**Ferritin**					
All (*n* = 1,070)	2.33 (*p* <0.001)	0.474 (p <0.001)	2.04 (*p* = 0.002)	-^‡^	0.33 (*p* <0.001)
Obese (*n* = 483)	1.96 (*p* = 0.046)	0.510 (*p* <0.001)	1.57 (*p* = 0.204)	2.62 (p <0.001)	0.51 (*p* = 0.010)
Non-obese (*n* = 587)	2.64 (*p* = 0.001)	0.456 (p <0.001)	2.47 (*p* = 0.003)	1.92 (*p* <0.001)	0.24 (*p* <0.001)
**D-dimer**					
All (*n* = 1,050)	2.17 (*p* <0.001)	0.022 (*p* = 0.676)	2.27 (p <0.001)	-^‡^	0.02 (*p* = 0.700)
*≥ 65 yrs* (*n* = 421)	2.00 (*p* = 0.005)	0.025 (*p* = 0.762)	2.13 (*p* = 0.003)	1.99 (*p* <0.001)	0.02 (*p* = 0.732)
* <65 yrs* (*n* = 629)	3.21 (*p* = 0.041)	0.025 (*p* = 0.718)	3.29 (*p* = 0.044)	2.97 (*p* <0.001)	0.02 (p=0.710)
**Creatinine**					
All (*n* = 1,084)	2.25 (*p* <0.001)	0.568 (*p* <0.001)	1.47 (*p* = 0.104)	2.44 (*p* <0.001)	0.57 (p <0.001)
**hs-Troponin T**					
All (*n* = 1,026)	2.25 (*p* <0.001)	0.194 (*p* <0.001)	1.99 (*p* = 0.003)	2.26 (*p* <0.001)	0.19 (*p* = 0.002)

* *Peak biomarker level was determined based on all measurements*.

*All values were natural log transformed and standardized for analysis*;

† *All models included terms for sex and were adjusted for age, obesity, race/ethnicity, and the number of biomarker measurements*.

*The outcome model included both sex and the biomarker as predictor variables*;

‡ *The outcome model included a biomarker by obesity interaction and therefore the main effect of the biomarker was not reported here*.

*OR, odds ratio; hs-CRP, high sensitivity C-reactive protein; hs-Troponin T, high sensitivity cardiac Troponin T*.

### Sensitivity Analyses

Additional analyses in the CUIMC/NYP COVID-19 Cohort tested the robustness of findings. In mediation analyses that adjusted for additional covariates and potential confounders (CAD, CKD, lung disease, hypertension, type 2 diabetes mellitus, cancer, heart failure, and stroke), the findings for each biomarker were highly consistent with the primary findings **(****Supplementary Table S7****)**. Similarly, in models that restricted data to patients that had complete data for all biomarkers and covariates (*N* = 1,688), mediation findings were also consistent with the main analyses **(****Supplementary Table S8****)**.

## Discussion

In this study of 4,017 patients with COVID-19 at two tertiary care centers, we confirm that compared to female sex, male sex was associated with higher mortality at 30 days. Further, we report the novel finding that specific biomarkers of pathophysiological processes mediate the effect of sex on COVID-19 outcomes. These include hs-cTnT, hs-CRP, D-dimer, ferritin and creatinine – with proportion mediated estimated to be greatest for cardiac injury (hs-cTnT), intermediate for inflammation (hs-CRP) and kidney function (creatinine), and least for thrombosis (D-dimer). Additional evaluated biomarkers were excluded because their peak values were not associated with sex (albumin, ALC, ESR, platelets), they were correlated and redundant with hs-CRP (WBC and automated lymphocytes) or because of excess missing data (IL-6 and lactate). Our findings suggest that biological pathways of inflammation, iron metabolism, and coagulation, as well as cardiac and kidney injury that may be downstream of these pathways, are implicated in the strong sex-related difference in COVID-19 outcomes.

Several prior small studies have looked at patterns of biomarker elevation by sex in association with COVID-19 outcomes, though none have performed rigorous analyses of these biomarkers as mediators. A study of 776 patients hospitalized in New Orleans with COVID-19 found that troponin and D-dimer were predictors of worse outcomes in men, while ferritin was associated with death only in women (16). In a retrospective review of 168 patients hospitalized with COVID-19 in Wuhan, China, there were five biomarkers identified that were higher among men who died than among women who died (NLR, CRP, AST, LDH, and creatinine) (9). Importantly, ours is the first study to report which biomarkers and pathways are potential causal mediators of sex effects. Our findings are robust even after adjusting for multiple baseline comorbidities, and our work is rigorous in providing replication of key findings at two independent major academic medical centers with large numbers of complex and severe COVID-19 cases.

Sex differences in inflammatory responses, as reflected by peak hs-CRP and ferritin, may amplify sex differences in cardiac and renal injury and thus contribute to our finding that biomarkers of cardiac and kidney end-organ damage mediate a substantial proportion of the effect of sex on COVID-19 outcomes. Although previous studies have identified sex differences in the rate of COVID-19-related myocardial injury (3) and an association between acute cardiac injury and death in COVID-19 (23) ours is the first to address cardiac injury as a mediator of sex effects on COVID-19 outcomes. Similarly, prior studies have demonstrated a sex difference in COVID-19-related kidney disease and acute kidney injury (24), yet biomarkers of renal function have never been tested as mediators. Further studies are required to determine the extent to which the effects of inflammation and cardiac and renal damage are independent contributors to sex-mediation of poor COVID-19 outcomes.

Of several markers (including WBC and ESR, see **Supplementary Table S2**), we focused on hs-CRP as representative of systemic inflammation and broad activation of innate and adaptive immunity in COVID-19. Prior studies, including our own (22), have demonstrated an association between elevated inflammatory markers and death or ICU admission, with a stronger association in men as compared to women (9, 15, 25). However, our study is the first to suggest that inflammation directly mediates the effect of sex on COVID-19-related mortality. This mediation could be due to established sex differences in both the innate and adaptive immune pathways (26). Many of the genes involved in innate and adaptive immunity are located on the X chromosome. Some of them may escape X inactivation, leading to a more comprehensive immune response in women as compared to men (26, 27). Men and women also differ in the production of cytokines and chemokines by innate immune cells. During inflammatory stress, men have higher levels of pro-inflammatory cytokine production than females (26, 28), which could lead to a more severe cytokine storm associated with worse COVID-19 outcomes. Apart from genetics, there may be hormonal factors contributing to sex differences in the immune response to infection (29, 30). Testosterone is known to have an immunosuppressive effect (11, 26). At the same time, estradiol is thought to enhance cell-mediated and humoral immune responses, and progesterone has anti-inflammatory effects and may also contribute to differences in T cell populations (26). In secondary analyses, we found a trend toward a greater mediation effect of hs-CRP in patients age <50 years, which might support a role for the greater hormonal differences at pre-menopausal age in the contribution to sex differences in the inflammatory milieu and COVID-19.

Ferritin was a less potent mediator than hs-CRP of the effect of sex on COVID-19 outcomes, though the mediation effect was statistically significant and may be clinically important. In studies of patients with COVID-19, ferritin has been shown to correlate with disease severity in both men and women (31–33). In one study and in contrast to our findings, ferritin levels were found to be independently associated with death in women but not in men (16). Our larger CUIMC/NYPH data is the first to suggest that ferritin mediates the effect of male sex on worse outcomes in COVID-19 and this finding was highly consistent in the independent MGH registry. There are multiple possible explanations for the role of ferritin in mediating sex-related outcomes. Ferritin synthesis may increase as a result of the COVID-19 cytokine storm or inflammation may stimulate leakage of intracellular ferritin (33). This would suggest that peak ferritin levels, similar to hs-CRP, reflect systemic inflammation in mediating the effect of sex on outcomes. Alternatively, ferritin may play an independent causal role in the inflammatory cascade, acting as a mediator of immune dysregulation (33). Given that women of all ages have lower ferritin levels than men (34), lower baseline levels of ferritin could in fact be protective in women. Interestingly, when stratified by obesity, more of the effect of male sex on the primary outcome of death was explained by peak ferritin among obese patients as compared to non-obese patients. This finding may relate to differences in significance of ferritin levels in non-obese vs. obese patients. While in lean individuals ferritin may predominantly be a marker of iron stores, in obesity ferritin may correlate more with inflammation (35).

In the primary CUIMC/NYP cohort, we found that peak D-dimer levels were also mediators of the effect of sex on COVID-19 outcomes. In contrast, D-dimer levels were not found to be a mediator of the association between sex and COVID-19 outcomes in the MGH cohort. One possible explanation is that this might reflect differences in anticoagulation practice patterns for patients with COVID-19 across institutions, particularly early in the pandemic (36). Published work does suggest that microvascular dysfunction and thrombosis play a role in the pathogenesis of COVID-19, and studies have demonstrated an increased risk of severe outcomes and death in patients with elevated D-dimer (37). While there have been insufficient data to demonstrate a sex difference in the association between D-dimer and outcomes, sex differences in endothelial dysfunction have been well-established (38). Increased coagulation disorders may also cause more myocardial injury in men than in women, leading to worse outcomes (3). Prior studies have suggested that menopause is a risk factor for worse outcomes in women with COVID-19 (13, 39). In our age-stratified secondary analyses (≥ 50 vs. <50 years of age) designed as a surrogate for pre- and post-menopausal status in women, the proportion mediated by D-dimer was similar across younger vs. older age strata suggesting that thromboembolic mechanisms that drive higher risk in men are unlikely to reflect hormonal differences found in pre- or post-menopausal women. Future studies will need to focus more specifically on sex-related COVID-19 risks, including thromboembolic, in pre- and post-menopausal women.

Methodological strengths of this work include the novel application of mediation analysis to sex-related COVID-19 outcomes as well as a robust replication sample in which findings were highly consistent. Additional strengths include the sample size, the inclusion of a large percentage of under-represented minorities and the evaluation of 15 distinct biomarkers as potential mediators of sex effects on COVID-19 outcomes. There are several limitations of our study. First, the study used observational data extracted from the EHR in which missing data and measurement error are inherent and can result in biased findings (40). Second, the causal mediation framework assumes no unmeasured confounding (21). Third, despite robust replication across CUIMC/NYP and MGH cohorts, these two studies have differences in design and regional clinical contexts. Fourth, biomarkers are inherently limited as a markers of causal mechanisms and in therapeutic targeting, as they are surrogates for the underlying causal pathway. Indeed, serum creatinine has significant limitations as a measure of kidney function or as a surrogate for kidney injury, but data were missing for calculation of more reliable measures of acute kidney injury (e.g., using KDIGO recommendations) (41). Further, all validated equations for eGFR incorporate sex rendering invalid analyses of sex mediation through such a derived biomarker. However, ease in clinical use of these biomarkers means our findings are immediately translatable to clinical practice. There are also redundancies between clinically available biomarkers, e.g., we selected hsCRP over WBC and ESR because hsCRP had stronger sex-mediation effects than the other inflammatory markers (**Supplementary Table S2**). Moreover, given the complex relationships among sex, mediators and other factors in severe COVID-19 outcomes, future work on additional pathophysiological pathways is needed. Our analyses were limited to the 15 clinically available candidate blood biomarkers selected for our study, and therefore we cannot exclude other biomarkers and organs as mediators. Specifically, our initial analyses suggest that larger sample sizes than ours are required to study the effect of biomarkers of hepatic injury within racial and ethnic strata. Finally, future studies are needed to define optimal clinical translation including use of mediators in clinical trials stratifying for high-risk patients.

In summary, we identified several distinct biomarkers of pathophysiological processes, including cardiac injury, that are reproducible mediators of the effect of sex on COVID-19 outcomes. Each of these pathways is a downstream manifestation of genetic, hormonal, and socio-demographic differences between men and women. And each offers a unique opportunity for better risk stratification, resource utilization, and targeted clinical trials toward personalized interventions and therapies for subgroups of patients at highest risk for poor COVID-19 outcomes.

## Data Availability Statement

The raw data supporting the conclusions of this article will be made available by the authors, without undue reservation.

## Ethics Statement

The studies involving human participants were reviewed and approved by the CUIMC Institutional Review Board (#AAAS9835) and the Partners HealthCare Institutional Review Board (#2020P000829). Written informed consent for participation was not required for this study in accordance with the national legislation and the institutional requirements.

## Author Contributions

HL: literature search and writing—original draft preparation. EK and CS: database organization and statistical analysis. HL, AF, and MR: conceptualization and writing—draft revision. All authors contributed to manuscript revision, provided comments, and agreed to the published version of the manuscript.

## Funding

Support for the design, conduct, and reporting of this research was provided by NIH R01 GM127862 to AF and R01 HL132561 and R01 HL113147 to MR.

## Conflict of Interest

AF receives sponsored research support from Bristol Myers Squibb/Pfizer and Fitbit and has received expert witness testimonial fees from Round Table Group. The remaining authors declare that the research was conducted in the absence of any commercial or financial relationships that could be construed as a potential conflict of interest.

## Publisher's Note

All claims expressed in this article are solely those of the authors and do not necessarily represent those of their affiliated organizations, or those of the publisher, the editors and the reviewers. Any product that may be evaluated in this article, or claim that may be made by its manufacturer, is not guaranteed or endorsed by the publisher.
