# Supplementary material for: Biomarkers of Cardiac Injury, Renal Injury, and Inflammation Are Strong Mediators of Sex-Associated Death in COVID-19
Source: Front Cardiovasc Med. 2022 Apr 25;9:809997. doi: 10.3389/fcvm.2022.809997 (PMC9081502; doi:10.3389/fcvm.2022.809997)
Supplement: Supplementary file 1 [file Data_Sheet_1.docx]

***Supplemental Material***

Table of Contents

[**Supplemental Table S1**. ICD-10-CM Codes 2](#_Toc80028388)

[**Supplemental Table S2**. Mediation analyses of peak values^*^ of excluded biomarkers in CUIMC/NYP COVID-19 cohort 3](#_Toc80028389)

[**Supplement Table S3.** Clinical characteristics of patients with complete data vs. missing data 5](#_Toc80028390)

[**Supplement Table S4.** Clinical characteristics and admission labs overall and by sex in the MGH cohort 6](#_Toc80028391)

[**Supplement Table S5.** Peak laboratory values and outcomes overall and by sex in the MGH cohort 9](#_Toc80028392)

[**Supplement Table S6**. Mediation analysis of peak values^*^ of biomarkers in the CUIMC/NYP COVID-19 cohort fully adjusted for additional comorbidity variables 10](#_Toc80028393)

[**Supplement Table S7.** Mediation analysis of peak values^*^ of biomarkers in the CUIMC/NYP COVID-19 cohort restricted to individuals with complete data for all variables (N=1,688) 11](#_Toc80028394)

[**Supplement Table S8.** Mediation analysis of peak values^*^ of biomarkers in the CUIMC/NYP COVID-19 cohort stratified by participants who are ≥ or < age 50 6](#_Toc80028395)

[**Supplement Table S9.**  Median and IQR for age overall and stratified by sex as well as distribution of participants aged ≥65 years or aged ≥50 years overall and by sex in the CUIMC/NYP COVID-19 cohort 12](#_Toc80028396)

# **Supplemental Table S1**. ICD-10-CM Codes

| **Comorbidity** | **ICD-10-CM Codes** |
| --- | --- |
| Hypertension | I10-13, I15-16, O10.1-10.4, O10.9 |
| Diabetes | E08-11, E13, O24.4 |
| Coronary artery disease | I21-25, Z98.61, Z95.1 |
| Heart failure | I09.81, I11.0, I13.0, I50, I42 |
| Chronic lung disease | J40-47, J60, J66, J67.2, J67.8-67.9, J68.4, J84, G47.3 |
| Stroke/transient ischemic attack | I60-64, I69, H34.1, G45 |
| Chronic kidney disease | N03, N07-08, N11, N14, N18-19, N29, I12, I13, Z99.2, E10.22, E11.22, E13.22, E08.22, O10.3, D63.1 |

# **Supplemental Table S2**. Mediation analyses of peak values^*^ of excluded biomarkers in CUIMC/NYP COVID-19 cohort

|  | Total Effect Model^†^  Outcome: Death | Mediator Model^†^  Outcome: biomarker | Outcome model^†^  Outcome: Death | | Proportion  Mediated |
| --- | --- | --- | --- | --- | --- |
|  | OR (sex, p) | Est (sex, p) | OR (sex, p) | OR (biomarker, p) | (p-value) |
| **White blood cell count (WBC)** |  |  |  |  |  |
| (n=2,112) | 2.00 (p<0.001) | 0.193 (p=0.003) | 1.84 (p<0.001) | -^‡^ | 0.22 (p<0.001) |
| *≥ 65 yrs* (n=1,109) | 1.95 (p<0.001) | 0.143 (p=0.018) | 1.85 (p<0.001) | -^‡^ | 0.17 (p=0.012) |
| *< 65 yrs* (n=1,003) | 2.08 (p=0.004) | 0.213 (p=0.002) | 1.82 (p=0.029) | 2.32 (p=0.011) | 0.27 (p=0.004) |
| *≥ 65&White* (n=140) | 3.12 (p=0.010) | -0.030 (p=0.863) | 3.23 (p=0.009) | 1.29 (p=0.197) | <0.01 (p=0.936) |
| *≥ 65&Black* (n=116) | 1.42 (p=0.432) | 0.131 (p=0.448) | 1.32 (p=0.567) | 3.35 (p<0.001) | 0.24 (p=0.510) |
| *≥ 65&Hisp* (n=548) | 2.45 (p<0.001) | 0.205 (p=0.018) | 2.27 (p<0.001) | 2.57 (p<0.001) | 0.19 (p=0.024) |
| *≥ 65&Other* (n=305) | 1.22 (p=0.431) | 0.101 (p=0.390) | 1.13 (p=0.662) | 2.84 (p<0.001) | 0.28 (p=0.490) |
| **Erythrocyte sedimentation rate** **(ESR)** |  |  |  |  |  |
| (n=1,869) | 1.95 (p<0.001) | 0.030 (p=0.523) | 1.95 (p<0.001) | -^‡^ | 0.02 (p=0.470) |
| **IL-6** |  |  |  |  |  |
| (n=1,240) | 1.85 (p<0.001) | 0.278 (p<0.001) | 1.51 (p=0.012) | -^‡^ | 0.49 (p<0.001) |
| *≥ 65 yrs* (n=674) | 1.85 (p=0.001) | 0.304 (p<0.001) | 1.48 (p=0.044) | 3.48 (p<0.001) | 0.49 (p<0.001) |
| *< 65 yrs* (n=566) | 1.86 (p=0.042) | 0.185 (p=0.057) | 1.72 (p=0.105) | 7.45 (p<0.001) | 0.41 (p=0.060) |
| **Platelet** |  |  |  |  |  |
| (n=2,112) | 2.00 (p<0.001) | -0.067 (p=0.131) | 1.94 (p<0.001) | 0.81 (p<0.001) | 0.02 (p=0.140) |
| **Lymphocytes (automated)** |  |  |  |  |  |
| (n=2,025) | 1.99 (p<0.001) | -0.257 (p<0.001) | 1.84 (p<0.001) | -^‡^ | 0.27 (p<0.001) |
| **Lactate** |  |  |  |  |  |
| (n=1,302) | 2.10 (p<0.001) | 0.206 (p<0.001) | 2.05 (p<0.001) | -^‡^ | 0.18 (p<0.001) |
| *≥ 65 yrs* (n=689) | 2.13 (p<0.001) | 0.194 (p=0.012) | 2.03 (p<0.001) | 2.06 (p<0.001) | 0.16 (p=0.008) |
| *< 65 yrs* (n=613) | 2.05 (p=0.007) | 0.177 (p=0.041) | 2.19 (p=0.009) | 2.96 (p<0.001) | 0.20 (p=0.040) |
|  |  |  |  |  |  |
| **Aspartate aminotransferase (AST)** |  |  |  |  |  |
| (n=2,080) | 1.99 (p<0.001) | 0.334 (p<0.001) | 1.66 (p<0.001) | -^‡^ | 0.35 (p<0.001) |
| *White* (n=195) | 1.92 (p=0.088) | 0.430 (p=0.002) | 1.17 (p=0.722) | 4.81 (p=0.002) | 0.77 (p=0.082) |
| *Black* (n=250) | 1.50 (p=0.247) | 0.408 (p=0.001) | 1.12 (p=0.755) | 2.29 (p<0.001) | 0.58 (p=0.250) |
| *Hispanic* (n=1,024) | 2.60 (p<0.001) | 0.413 (p<0.001) | 2.16 (p<0.001) | 1.98 (p<0.001) | 0.27 (p<0.001) |
| *Other* (n=611) | 1.44 (p=0.095) | 0.141 (p=0.090) | 1.40 (p=0.145) | 2.28 (p<0.001) | 0.24 (p=0.136) |
| **Alanine aminotransferase (ALT)** |  |  |  |  |  |
| (n=2,080) | 1.99 (p<0.001) | 0.418 (p<0.001) | 1.67 (p<0.001) | -^‡^ | 0.28 (p<0.001) |
| *White* (n=195) | 1.92 (p=0.088) | 0.530 (p<0.001) | 1.10 (p=0.813) | 3.20 (p<0.001) | 0.82 (p=0.092) |
| *Black* (n=250) | 1.50 (p=0.247) | 0.491 (p<0.001) | 1.19 (p=0.633) | 1.65 (p=0.004) | 0.43 (p=0.268) |
| *Hispanic* (n=1,024) | 2.60 (p<0.001) | 0.466 (p<0.001) | 2.27 (p<0.001) | 1.43 (p<0.001) | 0.17 (p<0.001) |
| *Other* (n=611) | 1.44 (p=0.095) | 0.274 (p=0.001) | 1.33 (p=0.203) | 1.65 (p<0.001) | 0.31 (p=0.058) |
| **Albumin** |  |  |  |  |  |
| (n=2,078) | 1.98 (p<0.001) | -0.018 (p=0.674) | 2.18 (p<0.001) | -^‡^ | <0.01 (p=0.720) |
| **Absolute lymphocyte count (ALC)** |  |  |  |  |  |
| (n=1,027) | 1.90 (p<0.001) | -0.043 (p=0.501) | 1.90 (p<0.001) | 1.05 (p=0.472) | <0.01 (p=0.720) |

*Peak biomarker level was determined based on all measurements. All values were natural log transformed and standardized for analysis; †All models included terms for sex and were adjusted for age, obesity, race/ethnicity, and the number of biomarker measurements. The outcome model included both sex and the biomarker as predictor variables; ‡ The outcome model included a biomarker by sex, age, obesity, and/or race/ethnicity interaction and therefore the main effect of the biomarker was not reported here. Significant interactions for each biomarker are WBC by age and race, ESR by age, IL-6 by age and obesity, lymphocytes by sex, obesity, and race/ethnicity, lactate by age, AST by race/ethnicity, ALT by race/ethnicity, and albumin by sex and age.

# **Supplement Table S3.** Clinical characteristics of patients with complete data vs. missing data

|  | CUIMC/NYP (N=2,626) | |
| --- | --- | --- |
|  | Complete Data^*^  (N=1,688) | Incomplete Data^*^  (N=938) |
| **Presentation to Care** |  |  |
| Age in years (Median [IQR]) | 65 (54,76)^‡^ | 68 (54,80)^‡^ |
| Age ≥ 65 years | 879/1688 (0.52)^‡^ | 541/938 (0.58)^‡^ |
| BMI (Median [IQR]) | 28.3 (24.8,32.8) | 27.2 (24,32.5) |
| White/non-Hispanic | 153/1688 (0.09) | 84/938 (0.09) |
| Black/non-Hispanic | 201/1688 (0.12) | 119/938 (0.13) |
| Hispanic | 831/1688 (0.49) | 483/938 (0.51) |
| Other | 503/1688 (0.30) | 252/938 (0.27) |
| **Co-morbidities** |  |  |
| Obesity^†^ | 643/1688 (0.38) | 151/938 (0.16) |
| Coronary artery disease/ myocardial infarction | 210/1688 (0.12) | 119/938 (0.13) |
| Hypertension | 936/1688 (0.55) | 494/938 (0.53) |
| Diabetes mellitus, type 2 | 648/1688 (0.38)^‡^ | 320/938 (0.34)^‡^ |
| Chronic Kidney Disease | 251/1688 (0.15) | 119/938 (0.13) |
| Lung Disease | 312/1688 (0.18) | 151/938 (0.16) |
| Cancer | 166/1688 (0.10) | 95/938 (0.10) |
| Heart Failure | 168/1688 (0.10) | 107/938 (0.11) |
| Stroke | 144/1688 (0.09) | 81/938 (0.09) |
| **30-Day Outcome** |  |  |
| Intensive care unit, ventilation or death | 677/1688 (0.40)^‡^ | 231/938 (0.25)^‡^ |
| Intensive care unit or ventilation | 498/1688 (0.30)^‡^ | 61/938 (0.07)^‡^ |
| Death | 406/1688 (0.24) | 217/938 (0.23) |

*Complete data includes patients with no missing information on peak CRP, peak D-dimer, peak Ferritin, peak Creatinine, peak Troponin, BMI, sex, age, race/ethnicity and outcome. Incomplete data includes patients missing information on at least one of these variables.

†Obesity is defined as BMI≥30

‡Proportion is statistically significantly different (p<0.05) between patients with complete data and patients with incomplete data.

# **Supplement Table S4.** Mediation analysis of peak values^*^ of biomarkers in the CUIMC/NYP COVID-19 cohort stratified by participants who are ≥ or < age 50

|  | Total Effect Model^†^  Outcome: Death | Mediator Model^†^  Outcome: biomarker | Outcome model^†^  Outcome: Death | | Proportion  Mediated |
| --- | --- | --- | --- | --- | --- |
|  | OR (sex, p) | Est (sex, p) | OR (sex, p) | OR (biomarker, p) | (p-value) |
| **hs-CRP** |  |  |  |  |  |
| All (n=1,978) | 2.00 (p<0.001) | 0.285 (p<0.001) | 1.71 (p<0.001) | 2.80 (p<0.001) | 0.42 (p<0.001) |
| *≥ 50 yrs* (n=1,648) | 2.01 (p<0.001) | 0.216 (p<0.001) | 1.72 (p<0.001) | 2.77 (p<0.001) | 0.35 (p<0.001) |
| *< 50 yrs* (n=330) | 2.91 (p=0.105) | 0.543 (p<0.001) | 2.56 (p=0.186) | 9.26 (p=0.100) | 0.59 (p=0.030) |
| **Ferritin** |  |  |  |  |  |
| (n=1,964) | 2.02 (p<0.001) | 0.539 (p<0.001) | 1.52 (p=0.001) | 2.09 (p<0.001) | 0.52 (p<0.001) |
| *≥ 50 yrs* (n=1,632) | 2.02 (p<0.001) | 0.406 (p<0.001) | 1.56 (p=0.001) | 1.99 (p<0.001) | 0.42 (p<0.001) |
| *< 50 yrs* (n=332) | 3.15 (p=0.083) | 1.097 (p<0.001) | 2.93 (p=0.218) | 7.82 (p<0.001) | 0.57 (p=0.014) |
| **D-dimer** |  |  |  |  |  |
| (n=1,814) | 2.03 (p<0.001) | 0.207 (p<0.001) | 1.84 (p<0.001) | 10.93 (p<0.001) | 0.22 (p<0.001) |
| *≥ 50 yrs* (n=1,500) | 2.03 (p<0.001) | 0.212 (p<0.001) | 1.85 (p<0.001) | 2.26 (p<0.001) | 0.22 (p<0.001) |
| *< 50 yrs* (n=314) | 2.92 (p=0.102) | 0.113 (p=0.367) | 2.55 (p=0.199) | 8.09 (p<0.001) | 0.19 (p=0.370) |
| **Creatinine** |  |  |  |  |  |
| (n=2,106) | 1.98 (p<0.001) | 0.430 (p<0.001) | 1.52 (p=0.001) | 2.24 (p<0.001) | 0.45 (p<0.001) |
| *≥ 50 yrs* (n=1,739) | 1.98 (p<0.001) | 0.429 (p<0.001) | 1.52 (p=0.002) | 2.24 (p<0.001) | 0.45 (p<0.001) |
| *< 50 yrs* (n=367) | 3.10 (p=0.082) | 0.438 (p<0.001) | 2.19 (p=0.241) | 2.54 (p<0.001) | 0.33 (p=0.062) |
| **hs-Troponin T** |  |  |  |  |  |
| (n=1,954) | 2.05 (p<0.001) | 0.301 (p<0.001) | 1.70 (p<0.001) | 2.55 (p<0.001) | 0.35 (p<0.001) |
| *≥ 50 yrs* (n=1,630) | 2.05 (p<0.001) | 0.316 (p<0.001) | 1.71 (p<0.001) | 2.45 (p<0.001) | 0.35 (p<0.001) |
| *< 50 yrs* (n=324) | 2.82 (p=0.113) | 0.233 (p=0.029) | 3.59 (p=0.125) | 5.17 (p<0.001) | 0.24 (p=0.058) |

^*^Peak biomarker level was determined based on all measurements. All values were natural log transformed and standardized for analysis; †All models included terms for sex and were adjusted for age, obesity, race/ethnicity, and the number of biomarker measurements. The outcome model included both sex and the biomarker as predictor variables.

hs-CRP, high sensitivity C-reactive protein; hs-cTNT, high sensitivity cardiac Troponin T

# **Supplement Table S5.** Clinical characteristics and admission labs overall and by sex in the MGH cohort

|  | Overall  (N=1391) | Men  (N=794) | Women  (N=596) | P-value^*^ |
| --- | --- | --- | --- | --- |
| **Presentation to Care** |  |  |  |  |
| Age in years (Median [IQR]) | 60 (45.5,73.4) | 59.5 (46.4,72.1) | 60.4 (44,75.1) | 0.462 |
| Age ≥ 65 years | 549/1390(0.39) | 302/794(0.38) | 247/596(0.41) | 0.218 |
| White/non-Hispanic | 550/1275(0.43) | 313/714(0.44) | 237/561(0.42) | 0.608 |
| Black/non-Hispanic | 149/1275(0.12) | 74/714(0.10) | 75/561(0.13) | 0.116 |
| Hispanic | 492/1275(0.39) | 282/714(0.39) | 210/561(0.37) | 0.488 |
| Other | 84/1275(0.07) | 45/714(0.06) | 39/561(0.07) | 0.726 |
| Fever | 845/1391(0.61) | 509/794(0.64) | 335/596(0.56) | 0.003 |
| BMI (Median [IQR]) | 29.1 (25.4,33.7) | 28.6 (25.2,32.9) | 29.8 (25.6,35.0) | 0.016 |
| On Statins | 545/1391(0.39) | 346/794(0.44) | 199/596(0.33) | <0.001 |
| On ACEi or ARBs | 322/1391(0.23) | 174/794(0.22) | 148/596(0.25) | 0.226 |
| **Co-morbidities**^†^ |  |  |  |  |
| Obesity^‡^ | 565/1249(0.45) | 294/703(0.42) | 271/546(0.5) | 0.007 |
| Coronary artery disease | 211/1391(0.15) | 146/794(0.18) | 65/596(0.11) | <0.001 |
| Hypertension | 697/1391(0.50) | 404/794(0.51) | 293/596(0.49) | 0.561 |
| Diabetes mellitus type 2 | 445/1391(0.32) | 270/794(0.34) | 175/596(0.29) | 0.075 |
| Chronic Kidney Disease | 233/1365(0.17) | 151/778(0.19) | 82/586(0.14) | 0.011 |
| Lung disease | 413/1388(0.30) | 217/792(0.27) | 196/595(0.33) | 0.030 |
| Cancer | 209/1364(0.15) | 117/776(0.15) | 92/587 (0.16) | 0.821 |
| Heart Failure | 153/1391(0.11) | 94/794(0.12) | 59/596(0.10) | 0.291 |
| Stroke | 88/1391(0.06) | 53/794(0.07) | 35/596(0.06) | 0.619 |
| **Admission labs**^§^ **(Median [IQR])** |  |  |  |  |
| CRP (mg/L; n=1202) | 72.6 (30.8,144) | 79.1 (35.8,148) | 63.8 (25.7,136) | <0.001 |
| Ferritin (ug/L; n=1203) | 512 (242,1030) | 641 (334,1230) | 388 (183,692) | <0.001 |
| Creatinine (mg/dL; n=1257) | 0.95 (0.78,1.28) | 1.05 (0.88,1.39) | 0.8 (0.67,1.06) | <0.001 |
| D-dimer (ng/mL; n=1148) | 1020 (658,1850) | 947 (628,1740) | 1120 (690,1950) | 0.005 |
| hs-Troponin T (ng/L; n=1168) | 11 (6,31) | 13 (6,35) | 9 (6,24.5) | <0.001 |

*P-values correspond to a two-sample test of proportions (for categorical variables) or Wilcoxon rank sum tests (for numeric variables) comparing corresponding characteristics of male versus female patients; †Co-morbidities – coronary artery disease (CAD), myocardial infarction (MI), hypertension, pulmonary disease history and type 2 diabetes mellitus (T2DM) – were manually extracted based on admission notes, problem lists from past medical history and history of present illness; ‡Obesity is defined as BMI ≥ 30; §Admission labs recorded within +/-3 days of hospital admission

IQR, interquartile range; ACEi, angiotensin-converting enzyme inhibitors; ARB, angiotensin receptor blockers; BMI, body mass index; ACE, angiotensin-converting enzyme; ARB, angiotensin receptor blocker; CRP, C-reactive protein; hs-Troponin T, high sensitivity cardiac Troponin T

# **Supplement Table S6.** Peak laboratory values and outcomes overall and by sex in the MGH cohort

|  | Overall  (N=1391) | Men  (N=794) | Women  (N=596) | P-value^*^ |
| --- | --- | --- | --- | --- |
| **Peak labs (Median [IQR])** |  |  |  |  |
| CRP (mg/L; n=1314) | 138 (63.8,245) | 146 (71.5,265) | 121 (52.2,209) | <0.001 |
| Ferritin (ug/L; n=1316) | 780 (369,1600) | 1020 (532,2000) | 554 (273,1100) | <0.001 |
| Creatinine (mg/dL; n=1354) | 1.06 (0.84,1.66) | 1.17 (0.94,2.03) | 0.88 (0.71,1.24) | <0.001 |
| D-dimer (ng/mL; n=1297) | 1750 (908,4060) | 1800 (926,4360) | 1650 (881,3560) | 0.130 |
| hs-Troponin T (ng/L; n=1287) | 15 (6,42) | 17 (7,50) | 12 (6,32) | <0.001 |
| **Complications within 28 days**^†^ |  |  |  |  |
| ARDS | 318/1391(0.23) | 210/794(0.26) | 108/596(0.18) | <0.001 |
| Liver dysfunction | 260/1391(0.19) | 165/794(0.21) | 95/596(0.16) | 0.026 |
| Acute renal injury/failure | 321/1391(0.23) | 224/794(0.28) | 97/596(0.16) | <0.001 |
| **Outcomes at 28 days**^‡^ |  |  |  |  |
| ICU or death | 505/1386(0.36) | 319/792(0.40) | 186/593(0.31) | 0.001 |
| ICU | 429/1386(0.31) | 276/792(0.35) | 153/593(0.26) | <0.001 |
| Death | 172/1391(0.12) | 115/794(0.14) | 57/596(0.10) | 0.007 |

*P-values correspond to a two-sample test of proportions (for categorical variables) or Wilcoxon rank sum tests (for numeric variables) comparing corresponding characteristics of male versus female patients; †Complications reported – Acute Respiratory Distress Syndrome (ARDS), liver dysfunction (as reflected by aspartate aminotransferase (AST) or alanine aminotransferase (ALT) >3 times the upper limit of normal) and acute renal injury/failure – occurred within 28 days of presentation to care.

IQR, interquartile range; CRP, C-reactive protein; hs-Troponin T, high sensitivity cardiac Troponin T

# **Supplement Table S7**. Mediation analysis of peak values^*^ of biomarkers in the CUIMC/NYP COVID-19 cohort fully adjusted for additional comorbidity variables

|  | Total Effect Model^†^  Outcome: Death | Mediator Model^†^  Outcome: biomarker | Outcome model^†^  Outcome: Death | | Proportion  Mediated |
| --- | --- | --- | --- | --- | --- |
|  | OR (sex, p) | Est (sex, p) | OR (sex, p) | OR (biomarker, p) | (p-value) |
| **hs-CRP** |  |  |  |  |  |
| All (n=1,978) | 2.03 (p<0.001) | 0.276 (p<0.001) | 1.75 (p<0.001) | 2.85 (p<0.001) | 0.41 (p<0.001) |
| *Obese* (n=748) | 2.06 (p=0.001) | 0.199 (p=0.003) | 1.95 (p=0.004) | 9.32 (p<0.001) | 0.40 (p=0.002) |
| *Not obese* (n=1,230) | 2.06 (p<0.001) | 0.322 (p<0.001) | 1.67 (p=0.002) | 2.88 (p<0.001) | 0.40 (p<0.001) |
| **Ferritin** |  |  |  |  |  |
| (n=1,964) | 2.03 (p<0.001) | 0.537 (p<0.001) | 1.53 (p=0.001) | 2.10 (p<0.001) | 0.53 (p<0.001) |
| *Obese* (n=748) | 2.10 (p<0.001) | 0.650 (p<0.001) | 1.37 (p=0.180) | 3.10 (p<0.001) | 0.71 (p<0.001) |
| *Not obese* (n=1,216) | 2.04 (p<0.001) | 0.472 (p<0.001) | 1.64 (p=0.003) | 2.11 (p<0.001) | 0.42 (p<0.001) |
| **D-dimer** |  |  |  |  |  |
| (n=1,814) | 2.06 (p<0.001) | 0.188 (p<0.001) | 1.89 (p<0.001) | 11.03 (p<0.001) | 0.20 (p<0.001) |
| *≥ 65 yrs* (n=945) | 1.92 (p<0.001) | 0.144 (p=0.025) | 1.83 (p<0.001) | 2.19 (p<0.001) | 0.16 (p=0.030) |
| *< 65 yrs* (n=869) | 2.49 (p=0.001) | 0.200 (p=0.006) | 2.25 (p=0.006) | 3.86 (p<0.001) | 0.25 (p=0.006) |
| **Creatinine** |  |  |  |  |  |
| (n=2,106) | 2.01 (p<0.001) | 0.420 (p<0.001) | 1.44 (p=0.005) | 2.74 (p<0.001) | 0.54 (p<0.001) |
| **hs-Troponin T** |  |  |  |  |  |
| (n=1,954) | 2.06 (p<0.001) | 0.276 (p<0.001) | 1.75 (p<0.001) | 2.75 (p<0.001) | 0.33 (p<0.001) |

*Peak biomarker level was determined based on all measurements. All values were natural log transformed and standardized for analysis; †All models included terms for sex and were adjusted for age, obesity, race/ethnicity, the number of biomarker measurements, coronary artery disease, chronic kidney disease, lung disease, hypertension type 2 diabetes mellitus, cancer, heart failure, and stroke. The outcome model included both sex and the biomarker as predictor variables.

OR, odds ratio ; hs-CRP, high sensitivity C-reactive protein; hs-cTNT, high sensitivity cardiac Troponin T

# **Supplement Table S8.** Mediation analysis of peak values^*^ of biomarkers in the CUIMC/NYP COVID-19 cohort restricted to individuals with complete data for all variables (N=1,688)

|  | Total Effect Model^†^  Outcome: Death | Mediator Model^†^  Outcome: biomarker | Outcome model^†^  Outcome: Death | | Proportion  Mediated |
| --- | --- | --- | --- | --- | --- |
|  | OR (sex, p) | Est (sex, p) | OR (sex, p) | OR (biomarker, p) | (p-value) |
| **hs-CRP** |  |  |  |  |  |
| All (n=1,688) | 2.09 (p<0.001) | 0.316 (p<0.001) | 1.78 (p<0.001) | 2.46 (p<0.001) | 0.40 (p<0.001) |
| *Obese* (n=643) | 2.33 (p=0.001) | 0.235 (p=0.001) | 2.15 (p=0.001) | 6.91 (p<0.001) | 0.38 (p=0.002) |
| *Not obese* (n=1,045) | 1.96 (p<0.001) | 0.362 (p<0.001) | 1.58 (p=0.009) | 2.49 (p<0.001) | 0.43 (p<0.001) |
| **Ferritin** |  |  |  |  |  |
| (n=1,688) | 2.09 (p<0.001) | 0.581 (p<0.001) | 1.52 (p=0.003) | 2.04 (p<0.001) | 0.53 (p<0.001) |
| *Obese* (n=643) | 2.33 (p<0.001) | 0.666 (p<0.001) | 1.52 (p=0.080) | 2.76 (p<0.001) | 0.63 (p<0.001) |
| *Not obese* (n=1,045) | 1.96 (p<0.001) | 0.516 (p<0.001) | 1.53 (p=0.014) | 2.03 (p<0.001) | 0.47 (p<0.001) |
| **D-dimer** |  |  |  |  |  |
| (n=1,688) | 2.09 (p<0.001) | 0.203 (p<0.001) | 1.91 (p<0.001) | 10.01 (p<0.001) | 0.21 (p<0.001) |
| *≥ 65 yrs* (n=879) | 1.97 (p<0.001) | 0.168 (p=0.011) | 1.84 (p<0.001) | 2.11 (p<0.001) | 0.17 (p=0.008) |
| *< 65 yrs* (n=809) | 2.36 (p=0.001) | 0.199 (p=0.009) | 2.14 (p=0.008) | 3.24 (p<0.001) | 0.24 (p=0.006) |
| **Creatinine** |  |  |  |  |  |
| (n=1,688) | 2.09 (p<0.001) | 0.438 (p<0.001) | 1.60 (p=0.001) | 2.15 (p<0.001) | 0.42 (p<0.001) |
| **hs-Troponin T** |  |  |  |  |  |
| (n=1,688) | 2.09 (p<0.001) | 0.275 (p<0.001) | 1.79 (p<0.001) | 2.59 (p<0.001) | 0.31 (p<0.001) |

*Peak biomarker level was determined based on all measurements. All values were natural log transformed and standardized for analysis; †All models included terms for sex and were adjusted for age, obesity, race/ethnicity, and the number of biomarker measurements. The outcome model included both sex and the biomarker as predictor variables.

OR, odds ratio ; hs-CRP, high sensitivity C-reactive protein; hs-cTNT, high sensitivity cardiac Troponin T

# **Supplement Table S9.** Median and IQR for age overall and stratified by sex as well as distribution of participants aged ≥65 years or aged ≥50 years overall and by sex in the CUIMC/NYP COVID-19 cohort

|  | Overall  (N=2626) | Men  (N=1497) | Women  (N=1129) | P-value for test of  Male vs. Female |
| --- | --- | --- | --- | --- |
| Age in years (Median [IQR]) | 66 (54, 77) | 64 (53, 75) | 69 (57, 80) | <0.001 |
| Age ≥65 years | 1420/2626 (0.54) | 748/1497 (0.50) | 672/1129 (0.60) | <0.001 |
| Age ≥50 years | 2164/2626 (0.82) | 1203/1497 (0.80) | 961/1129 (0.85) | 0.002 |
